# Exploring Prehospital Data for Pandemic Preparedness: A Western Brazilian Amazon Case Study on COVID-19

**DOI:** 10.3390/ijerph21091229

**Published:** 2024-09-18

**Authors:** Eduardo Fernandes, Bernardo Maia da Silva, Cássia da Luz Goulart, Jefferson Valente, Nádia Cubas-Vega, Camila Sato, Anna Gabriela Rezende, Taynna Vernalha Rocha Almeida, Robson Luís Oliveira de Amorim, Jorge Luis Salinas, Wuelton Marcelo Monteiro, Guilherme Peixoto Tinoco Arêas, Fernando Almeida-Val

**Affiliations:** 1 Escola Superior de Ciências da Saúde, Universidade do Estado do Amazonas, Manaus 69050-010, Brazil; eduardo_fernandes@me.com (E.F.); jefferson.valente@yahoo.com.br (J.V.); milasuemi@yahoo.com.br (C.S.); wueltonmm@gmail.com (W.M.M.); 2 Fundação de Medicina Tropical Doutor Heitor Vieira Dourado, Manaus 69040-000, Brazil; bernardo.mpesq88@gmail.com (B.M.d.S.); cassiadaluzgoulart@gmail.com (C.d.L.G.); 3 Universidad Nacional Autónoma de Honduras, Tegucigalpa 11100, Honduras; nadiadr13@gmail.com; 4 Hospital de Pronto Socorro 28 de Agosto, Manaus 69057-000, Brazil; 5 Postgraduate Program in Health Sciences, Universidade Federal do Amazonas, Manaus 69000-000, Brazil; annagabrielarezende@gmail.com (A.G.R.); amorim.robson@gmail.com (R.L.O.d.A.); guilhermepta@ufam.edu.br (G.P.T.A.); 6 Ministério da Saúde, Brasilia 70756-550, Brazil; taynnavra@gmail.com; 7 Division of Infectious Diseases & Geographic Medicine, Stanford University, Stanford, CA 94305, USA; jlsalinas7@gmail.com

**Keywords:** prehospital care, emergency medical service, severe acute respiratory syndrome, COVID-19

## Abstract

Background: The timely management of rapidly evolving epidemiological scenarios caused by disease outbreaks is crucial to prevent devastating consequences. However, delayed laboratory diagnostics can hamper swift health policy and epidemic response, especially in remote regions such as the western Brazilian Amazon. The aim of the article is to analyze the impact of the COVID-19 pandemic on the volume and characteristics of emergency medical services (EMS) in Manaus, focusing on how the pandemic affected sensitive indicators such as response time and the use of advanced life support ambulances. Additionally, the study seeks to understand how changes in prehospital EMS patterns, triggered by the pandemic, could be utilized as health surveillance tools, enabling a more rapid response in epidemic scenarios. Methods: This retrospective, descriptive study included data from the SAMU (Serviço de Atendimento Móvel de Urgência) medical records between January and June 2020. Results: A total of 45,581 calls resulted in mobile units being dispatched during this period. These patients were predominantly male (28,227, 61.9%), with a median age of 47 years (IQR 30–67). The median response time significantly increased during the pandemic, reaching a median of 45.9 min (IQR 30.6–67.7) (*p* < 0.001). EMS calls were reduced for trauma patients and increased for other medical emergencies, especially respiratory conditions, concomitantly to an escalation in the number of deaths caused by SARS and COVID-19 (*p* < 0.001). The employment of advanced life support ambulances was higher during the pandemic phase (*p* = 0.0007). Conclusion: The COVID-19 pandemic resulted in a temporary disorder in the volume and reason for EMS calls in Manaus. Consequently, sensitive indicators like the response time and the employment of advanced life support ambulances were negatively affected. Sudden prehospital EMS pattern changes could play an important role in health surveillance systems, allowing for earlier establishment of countermeasures in epidemics. The impact of the COVID-19 pandemic on prehospital EMS and its role in health surveillance should be further explored.

## 1. Introduction

In Brazil, the prehospital emergency medical service (EMS) is called Serviço de Atendimento Móvel de Urgência (SAMU). It is a state-funded service, universal, and without charge for all inhabitants of the country, covering approximately 85.6% of the Brazilian population [1]. One of its main roles is to triage emergency calls according to their urgency, dispatch accordingly (ambulance, motorcycle, boat, or helicopter), and appoint to the most suitable medical center at the time [2]. The dynamics of the EMS system involve several key components that work together to provide timely and effective prehospital care. These include the dispatch center, response time, resource allocation (such as ambulances with basic or advanced life support), communication systems, and the coordination between EMS teams and healthcare facilities. 

Since the first cases of SARS-CoV-2, reported in December 2019 as a respiratory syndrome, COVID-19 has rapidly spread, becoming a health problem of global dimensions [3,4,5,6,7,8,9,10,11]. In March 2020, SARS-CoV-2 hit Brazil and affected a diversity of health services, including the prehospital EMS [12,13,14,15]. Data on severe acute respiratory syndrome (SARS) from the health surveillance system and the number of funerals indicate that, in Manaus, the first wave of the pandemic occurred in March 2020, reaching its peak in May 2020 [16,17]. The city of Manaus, the capital of the State of Amazonas, has a population of 2.06 million inhabitants according to the 2022 census [18]. This is equivalent to 52% of the population of the state of Amazonas (3.94 million). According to the Rosemary Costa Pinto Health Surveillance Foundation (FVS-RCP), the number of accumulated COVID-19 cases in Manaus, until 30 August 2023, surpassed 637,000 and 14,482 deaths [19].

In Manaus, the prehospital EMS covers over 2 million people distributed in urban, rural, and riverine areas. The Brazilian health emergency line is toll-free (#192) and reaches a local regulation center, where a specially trained team of operators, nurses, and physicians process the requests. The EMS physicians assess and classify requests according to their severity and into different syndromic diagnoses before dispatching emergency vehicles, when indicated [1,2].

During the pandemic, emergency services were compelled to adapt in response to the steep rise in the number of critical patients and the ensuing insufficiency of medical supplies and healthcare workers. The prehospital EMS faced changes in the volume of calls, non-transport rates, and out-of-hospital mortality patterns [20,21,22].

The aim of this article is to analyze the impact of the COVID-19 pandemic on the volume and characteristics of emergency medical services (EMS) in Manaus, focusing on how the pandemic affected sensitive indicators such as response time and the use of advanced life support ambulances. Additionally, this study seeks to understand how changes in prehospital EMS patterns, triggered by the pandemic, could be utilized as health surveillance tools, enabling a more rapid response in epidemic scenarios.

## 2. Materials and Methods

### 2.1. Study Settings and Patients

This was a retrospective study of all prehospital EMS calls received in Manaus from January to June 2020, which includes the first epidemic peak of the COVID-19 pandemic in Brazil. Patients included those of any age and gender, who were treated by EMS in Manaus and who were removed to a public or private emergency care unit (prehospital emergency care services and emergency hospitals). Nonurgent cases (those who received medical advice with no mobile unit being dispatched) were not included in this study. Response time is considered as the time elapsed from the initial phone contact until the arrival of the mobile unit at the scene. Medical assessments were grouped into 15 different categories as the main reasons for EMS calls from January to June 2020 (Table 1).

Data were also obtained regarding death secondary to an acute respiratory syndrome (including at least two of the following: fever, chills, sore throat, headache, cough, coryza, taste/smell disorder), including COVID-19, complicated with or because of severe acute respiratory syndrome (SARS) that is defined as at least one of dyspnea, chest pressure, cyanosis, or pulse oximetry < 95% while breathing room air. Subjects were characterized by gender, age, location, the reason for the EMS call, and response time (from first contact with the regulation center until the arrival of the mobile unit). Results were arranged in two different ways: in six clusters of epidemiological weeks—corresponding approximately to the first six months of 2020—and in two periods of time (pre-pandemic and pandemic), separated by the date 13 March 2020, when the first COVID-19 case was officially confirmed in the Amazon state.

### 2.2. Data Collection

Data from three different databases were used in this study: the prehospital EMS (SAMU, Manaus), the death information system (Sistema de Informação sobre Mortalidade; SIM), and the flu surveillance system (Sistema de Informação da Vigilância Epidemiológica da Gripe—SIVEP-Gripe; administered by the FVS-RCP). The SIM includes general data from death certificates. The SIVEP-Gripe consists of data from patients admitted with and/or deceased by SARS, such as COVID-19 confirmation details.

First, data were extracted from the EMS electronic database. Records on the variable “reasons for contact” with incomplete information were excluded. Next, from the SIM and SIVEP-Gripe databases, variables about death, age, sex, race, skin color, education, and underlying cause were analyzed. The applied filters also aimed to isolate the city of interest (Manaus) and the study period. Finally, data on reasons for contacts and deaths were grouped, and their respective totals were organized into epidemiological weeks so that a graphical representation was possible. The data were assessed between July and September 2023.

### 2.3. Data Analysis

COVID-19 data were arranged in epidemiological weeks, which are weeks with specific counts (generally from Sunday to Saturday), aiming to standardize, optimize, and compare epidemiological data [23]. Data related to SARS-related deaths and reasons for contact were analyzed within a specific period (first semester, 2020). The database was organized based on dates, and relevant variables were selected. Data were grouped by epidemiological week and reason for contact (or cause of death) to calculate frequencies and percentages. Data were presented in terms of percentages and means with respective deviations or medians accompanied by their interquartile ranges. For the comparison of proportions, chi-square or Fisher’s exact tests were used. For comparison of means, the t-test was performed and, when applicable, the Wilcoxon test or the Kruskal Wallis test were used as well. For multivariate analyses, generalized linear log-binomial models were used to estimate relative risk, and respective confidence intervals were set at the 95% level. For statistical analyses and formulation of figures, Stata 16.0 and R 4.3.0 were used.

## 3. Results

**Figure 1:**
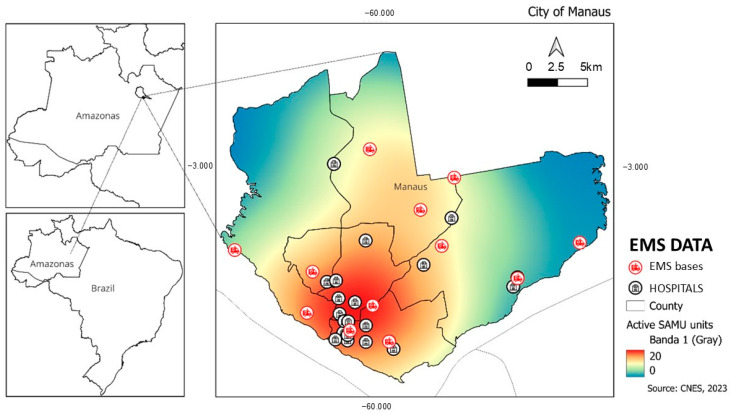
Distribution of hospitals, prehospital EMS bases, and variations in the displacement of mobile units during the study period (scale from blue to red), in Manaus.

The prehospital EMS coverage is higher in the city downtown (Figure 1), leading to faster response time occurring not only in areas with higher income but also in areas with elevated population concentration.

**Figure 2:**
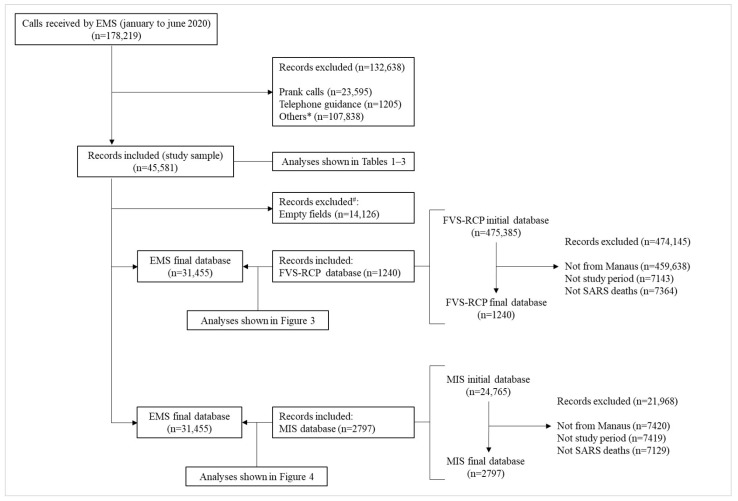
Flowchart of databases analyzed in the study: SAMU (EMS) database (respiratory emergencies), FVS-RCP, and SIM databases (confirmed COVID-19 cases and deaths). * Others: mistaken calls, calls to another destination (193, 190, which are numbers for the fire department and police, respectively), and dropped calls not regulated by the doctor; information from MRAO (medical regulation assistant operator); #: empty fields in the “reason for contact” variable were excluded from the SAMU database for adequacy and standardization in comparison with the FVS-RCP and SIM databases.

A total of 178,219 calls were received by the SAMU in Manaus between January and June 2020. Records of prank calls (*n* = 23,595), telephone guidance (*n* = 1205), and other reasons (mistaken, to other destinations, or resolved with guidance calls; *n* = 107,838) were excluded, resulting in 45,581 records of calls that resulted in mobile units dispatched for emergency assistance, which represent the study sample. Records from cities other than Manaus, including different time intervals and non-SARS cases, and their related outcomes, were excluded from the FVS-RCP and SIM databases. The empty fields in the “reason for contact” variable from the prehospital EMS database were excluded for adequacy, and standardization in comparison analyses was carried out with the other two databases. Details of the data management of the study databases are shown in Figure 2.

**Figure 3:**
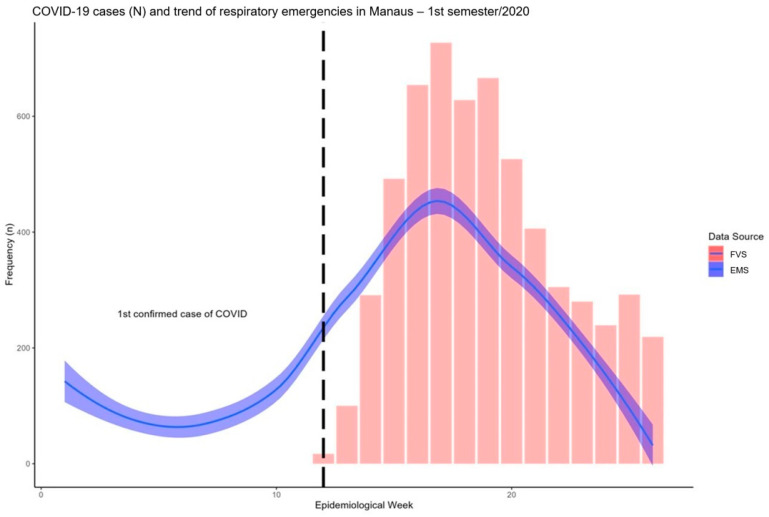
COVID-19 cases (in red) and trend (in blue) for respiratory emergency calls registered in the study period (the black dashed line indicates the date of the first official COVID-19 diagnosis in Manaus). The Loess method for curve smoothing was used (30% confidence area).

In Figure 3, COVID-19 cases and the trend for respiratory emergency calls registered in the study period are depicted. As shown, the number of respiratory emergency calls started to rise before the diagnosis of the first COVID-19 case in the city.

**Figure 4:**
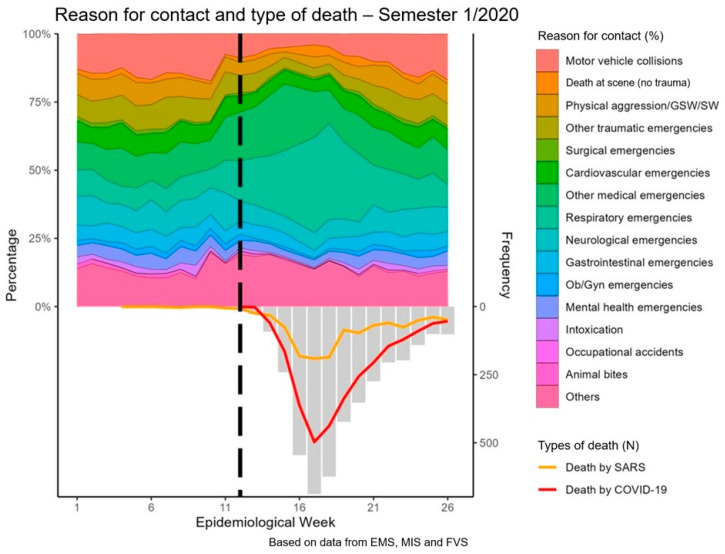
Impact of the pandemic on reasons for EMS calls over the first six months of 2020. The black dashed line indicates the date of the first official COVID-19 diagnosis in Manaus, and the gray bars indicate the cumulative total deaths from SARS and COVID-19.

The impact of the pandemic on the reasons for EMS calls indicates a reduction in calls for trauma patients and other external causes, whereas a proportional increase in medical emergencies was seen, especially calls for respiratory emergencies (*p* < 0.001). This period of asymmetry in the prehospital EMS cases correlates to an escalation in the number of deaths by SARS and COVID-19 in the city (bottom of Figure 4).

**Figure 5:**
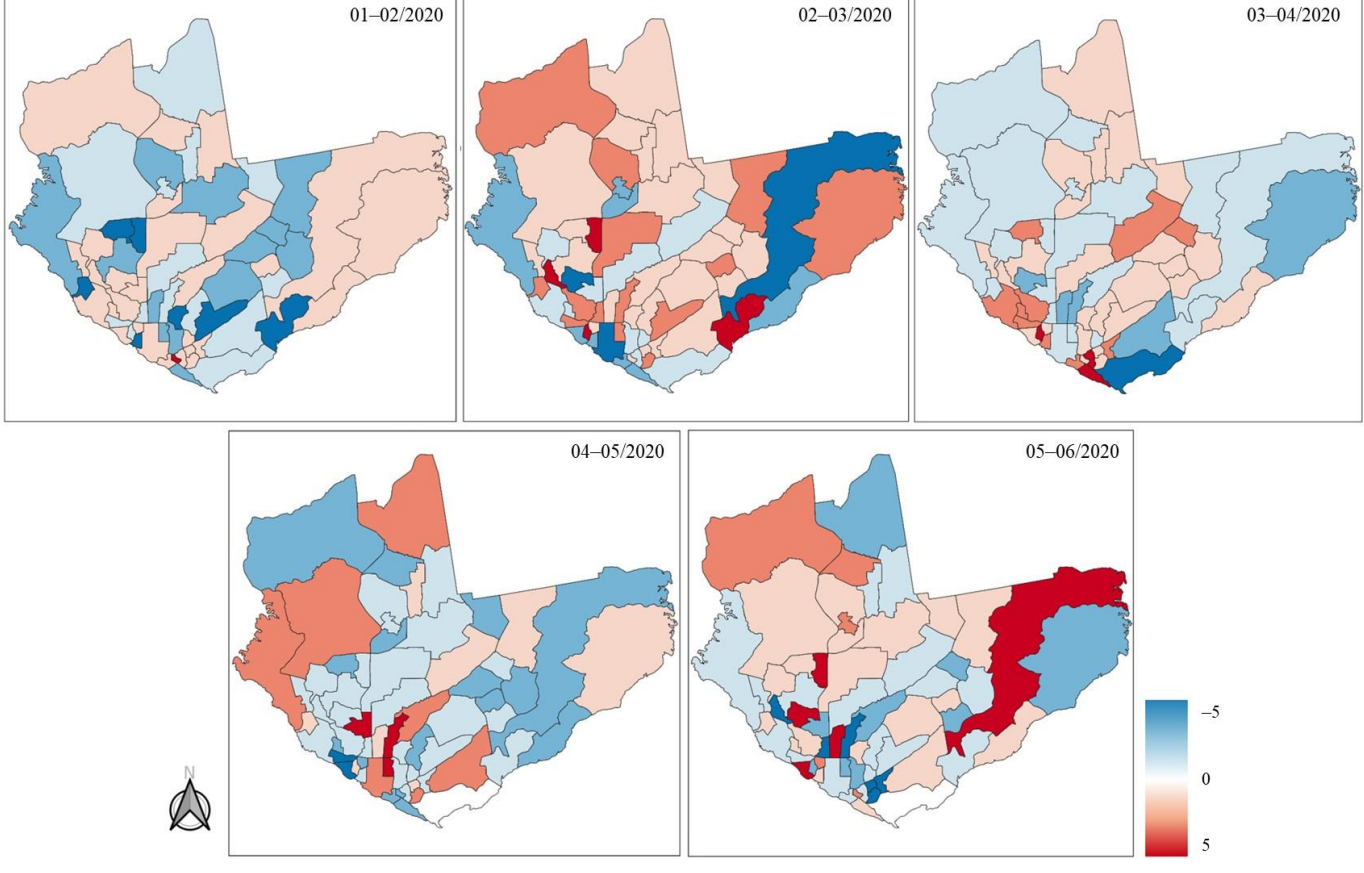
Heatmaps depict the rapid increase and decrease in the number of patients with respiratory emergencies in Manaus, month by month.

The number of EMS calls varied greatly in the first six months of 2020. Figure 5 depicts a delta calculation of the number of EMS calls between consecutive months in the Manaus municipality according to city region. The heatmaps show a rapid increase and decrease in the number of EMS calls addressing respiratory emergencies transferred by the EMS in Manaus during January–February, February–March, March–April, April–May, and May–June 2020 (Figure 5).

Notably, there was an increase in emergency calls from the city outskirts over time, showing an elevation of calls from the outer city over time and showing an elevation of calls from downtown distant locations and SAMU dispatch concentration units, which led to longer response times in these locations.

Patients assisted by the prehospital EMS in Manaus were predominantly male (28.227, 61.9%). The median age was 47 (IQR 30–67) years, reaching a higher median value (52, IQR 35–70) between epidemiological weeks 15 and 18, when the highest number of COVID-19 cases was confirmed in Manaus (*p* < 0.001). During this period, the number of dispatched units nearly doubled from baseline, with its highest in April 2020, accompanied by a significant deterioration in response time (*p* < 0.001) (Table 2).

A comparative analysis before and during the pandemic revealed a rise in the median age of patients served by EMS to 48 years (IQR 30–68 years), which was statistically significant (*p* < 0.001) (Table 3). In addition, a significant variation in the types of dispatched units was observed, with a higher proportion of advanced life support (ALS) units—mobile intensive care units—being employed in the pandemic phase (*p* = 0.0007). The analysis also clearly points to a reduction in motor vehicle collisions and other types of traumatic events in the pandemic period, alongside an expected increase in medical emergencies, especially respiratory cases (*p* < 0.001), as expected. Calls for cardiovascular events, on the other hand, were slightly reduced during the pandemic. Ultimately, response time was significantly compromised with the advent of the pandemic, with a significant increase in the median of time elapsed between the first contact with the EMS and the arrival of the mobile unit (*p* < 0.001) (Table 3).

## 4. Discussion

Our data have shown that the COVID-19 pandemic significantly affected various aspects of the prehospital EMS in Manaus, Brazil. The COVID-19 pandemic has profoundly impacted the prehospital EMS in Manaus, Brazil, altering the patient demographic to an older population requiring more advanced life support. During the pandemic, there was a significant increase in the median age of patients and a higher demand for advanced life support units, indicating a surge in critical cases.

In the United States, Yang et al. (2020) [24] described the first months of 2020, with 147 respiratory cases of prehospital EMS assistance. The average age was 75.7 years; 53.2% were female patients, and 45.1% of patients lived in long-term care facilities. In this population, 29.3% were asymptomatic and 53.6% had oxygen saturation less than or equal to 92% at room air. During follow-up until June 2020, mortality in the study population had reached 52.4%.

However, the surge in fatalities during the initial phase might not solely be ascribed to COVID-19. Indirect ramifications from restrictive measures and inadequate awareness about the disease possibly contributed. An association has already been reported between the beginning of the pandemic and a decrease in the number of EMS calls for cardiac emergencies and strokes, along with an increase in the number of calls for respiratory concerns and cardiac arrests, irrespective of actual COVID-19 incidence [20] Another North American experience described an increased number of prehospital deaths, associated with a decrease in the number of emergency department visits for myocardial infarction and stroke [25]. A similar scenario has now been depicted for Manaus in this study.

During the first half of 2020, we observed a significant reduction in the number of calls for trauma care (motor vehicle collisions, gunshot wounds, stab wounds, and other types of trauma). In part, these numbers probably reflect a change in the behavior of the population, secondary to restrictive sanctions, which reduced the flow of vehicles and contact between people outside their homes. Moreover, the substantial rise in calls for respiratory emergencies led SAMU Manaus to reach its operational capacity multiple times during the initial wave of the pandemic. The full occupation of vehicles prevented units from being sent to other types of calls, which may have contributed to the reduced assistance for cardiac, neurological, gynecological-obstetric, and mental health emergencies.

Besides, with the spread of SARS-CoV-2, EMS healthcare professionals (physicians, nurses, technicians, ambulance drivers) began sick leave. During that period, due to limited information about the disease, any professional displaying flu-like symptoms was required to stay away from work for a minimum of 15 days, even without laboratory confirmation. Testing was reserved for patients after hospitalization or in cases of death due to the scarcity of available tests. Furthermore, employees aged 60 and over and those with comorbidities were required to stay home as a precaution. Finally, those who were admitted with COVID-19 and some who eventually died were added to the list, particularly when the number of EMS calls was soaring, with the highest demand for ambulances. Staff shortage led to a dire reduction of the EMS operational capacity, not to mention the burning out of those who remained in service. Therefore, it is reasonable to admit that the demand for EMS in Manaus overreached its full capacity less than a month after the confirmation of the first COVID-19 case in the city.

This may have affected one of the main indicators of quality in prehospital services: response time. Arriving early in an emergency scene is a priority aspect of the service, as it deals with victims of severe trauma and time-sensitive clinical conditions, such as acute coronary syndromes, cerebrovascular syndromes, and cardiac arrests, among others. To meet this requirement, decentralization and strategical positioning of bases is recommended to provide adequate coverage in a reasonable time [26] and to avoid worse clinical outcomes and deaths.

In Manaus, response time reached a median of 45.9 min during the harshest period of the analysis. A few factors may have played a part. Local safety standards demanded terminal cleaning of the ambulance and all its equipment, which used to last approximately 30 min, after transporting a patient with SARS. Personal protective equipment (PPE) was essential for EMS teams during the pandemic, but donning and doffing gravely affected response time. Furthermore, the heat was extremely uncomfortable, as PPE was tightly sealed and prevented air circulation. In Manaus, the weather is extreme, with temperatures commonly rising above 35 °C throughout the day and high relative humidity, usually above 80%. Performing CPR, intubating, and transporting the patient while wearing PPE was challenging, both physically and mentally. Communication was very difficult, as the masks also filtered every attempt to communicate within the ambulance space and the regulation center, via a radio system. This was yet another factor contributing to healthcare personnel exhaustion and a risk to patient care.

Another important point is the period of the year when the pandemic started, which conflicted with the Amazon “winter”, the rainy season. When transporting patients with SARS, as a precaution against aerosols, all ambulance windows were kept open, the air conditioning was off, and the exhaust system was on. However, heavy rains turned ambulances inoperative, as windows could not be opened, PPE could not be wet (compromising protection), and the risk of falls was detrimental. Consequently, in the event of heavy rain, patients with SARS were informed that ground and boat ambulances were only sent off in appropriate weather conditions, assuring safe transportation. This factor also needs to be considered when analyzing response times during the pandemic.

The effect of the COVID-19 pandemic on the prehospital chain of survival was investigated in a recent meta-analysis. During the pandemic, response time was significantly higher, out-of-hospital cardiac arrests were more common and bystander defibrillation was significantly lower, indicating that prehospital processes were affected [27]. Unfortunately, this was no exception for the local EMS. In Manaus, emergency departments in public and private hospitals eventually ran out of beds, non-rebreathing masks, mechanical ventilators, and even oxygen. Ambulances were out of operation after leaving patients in hospitals with stretchers, masks, and oxygen tanks, unsure of when the essential material would be allowed to leave, frequently after removal to the mortuary.

EMS physicians working in the regulation center, responsible for assessing patients and dispatching ambulances, were overwhelmed not only by the exceptional number of calls but mostly by the hideous need to decide between circumstances: to send or not to send an ambulance? Also, would it arrive too late? Which hospital would be ready to provide minimally safe care at this time? Perhaps for these reasons, prehospital EMS faced an unprecedented increase in the rate of nontransports. Satty et al. (2021) [21] pointed out that, from March to May 2020, there was a 26.5% reduction in the volume of EMS calls in western Pennsylvania, USA, in addition to a 46.6% increase in nontransports (when a dispatched ambulance does not end in transport to a hospital), when compared to the same period of the year in the last 4 years before the pandemic.

The value of assistance by prehospital EMS is indisputable. However, their role as sentinel services has also been studied, aiding conventional health surveillance systems, especially in low- and middle-income countries. Friedman et al. (2020) [22] assessed how prehospital EMS data can be useful as sentinels within the surveillance system. The results were obtained by comparing clinical and sociodemographic variables between the pandemic period and the preceding years. An increase of 236.5% in respiratory cases, an increase of 145% out-of-hospital mortality, and a drop in SpO2 (average 77.7%) were identified. The highest out-of-hospital death rates were observed in low socioeconomic status areas, even though more respiratory cases were identified in higher socioeconomic regions.

Another use for EMS has also been postulated. Jaffe et al. (2020) [28] shared the experience of the Israeli emergency medical service, which is dedicated to tele-triage patients with suspected cases of COVID-19. Between February and March 2020, the volume of calls more than tripled, with the majority being cases of respiratory syndrome. These were home-screened by special teams that carried out local testing.

Although the EMS coverage in Brazil has grown in the last years, reaching approximately 85% of the population in 2019, inefficient coverage persists, especially in the northern region of the country, where the Amazon is located. Combined with this, inequality in the distribution of resources goes on, compromising both the response time and the resolution of the Brazilian EMS [29]. Comprehension of these changes might sustain better-planned actions in the setting of future pandemics and greater resilience, a fundamental aspect for EMS personnel [30,31].

This study has several limitations that warrant consideration. Firstly, the observational nature of our research design limits our ability to draw causal inferences from the data. Secondly, potential confounders could not be entirely ruled out, which might have influenced the outcomes. Thirdly, as the study was conducted in a specific geographic location, the findings may not be generalizable to other settings or populations. Additionally, the retrospective collection of data may be subject to selection and information biases, which could affect the accuracy of the results.

## 5. Conclusions

The COVID-19 pandemic resulted in a temporary disorder in the volume and reason for EMS calls in Manaus. Consequently, sensitive indicators such as the response time and the employment of advanced life support ambulances were negatively affected. Sudden prehospital EMS pattern changes could play an important role in health surveillance systems, allowing for earlier establishment of countermeasures in epidemics. Further research on the impact of the COVID-19 pandemic on prehospital EMS and its use as a sentinel for future epidemic scenarios should be fostered.

## Figures and Tables

**Table 1:** Description of diagnostic categories used by the physicians of the EMS regulation center in Manaus.

Categories	Main Associated Terms
Motor vehicle collisions	Collision, run over, rollover, motorcycle, car, bus, bike, truck
Death at scene (nontraumatic)	Death without assistance, death, sudden death
Aggresion/stab wound/gunshot wound	Firearm, stab wound, aggression against women, aggression against elderly, sexual assault
Other trauma	Building collapse, landslide, explosion, drowning, burn, electric shock
Surgical emergencies	Acute abdominal pain, postoperative complication
Cardiac emergencies	Syncope, chest pain, tachycardia, hypotension, hypertensive emergency
Other medical emergencies	Pain, edema, epistaxis, hemorrhage, hyperglycemia, hypothermia, infection, eye complaints, neoplasm, hypoactivity, bleeding
Respiratory emergencies	Respiratory failure, respiratory arrest, flu, suspected COVID-19, dyspnea, asthma, pneumonia, SARS
Neurological emergencies	Facial asymmetry, coma, stroke, headache, paralysis, seizure, hemiplegia, hemiparesis, altered mental status
Obstetric/gynecology emergencies	Miscarriage, amniorrhexis, eclampsia, preeclampsia, hyperemesis, out-of-hospital delivery, labor, vaginal bleeding
Mental health emergencies	Psychomotor agitation, hallucination, delirium, psychosis, panic attack, suicide attempt, hanging, self-harm
Intoxication	Alcohol, drugs, exogenous intoxication, toxic
Occupational accident	Work-related accident
Animal bites	Venomous animal accident, animal bite

**Table 2:** Characteristics of transports by the prehospital EMS in Manaus from January to June 2020 (epidemiological weeks 1 to 27).

	Total	Weeks 1–5	Weeks 6–9	Weeks 10–14	Weeks 15–18	Weeks 19–22	Weeks 23–27	*p*-Value
	*n* = 45,581	*n* = 6016	*n* = 5697	*n* = 7333	*n* = 12,600	*n* = 7960	*n* = 5975	
**Gender**								**0.002**
Female	17,354/45,581 (38.1%)	2233/6016 (37.1%)	2056/5697 (36.1%)	2785/7333 (38.0%)	4876/12,600 (38.7%)	3197/7960 (40.2%)	2207/5975 (36.9%)	
Male	28,227/45,581 (61.9%)	3783/6016 (62.9%)	3641/5697 (63.9%)	4548/7333 (62.0%)	7724/12,600 (61.3%)	4763/7960 (59.8%)	3768/5975 (63.1%)	
**Age (years)**	47.0 (30.0–67.0)	45.0 (28.0–67.0)	44.0 (27.0–67.0)	43.0 (26.0–64.0)	52.0 (35.0–70.0)	47.0 (30.0–67.0)	43.0 (27.0–65.0)	**<0.001**
**Dispatched unit**								0.82
Boat ambulance	63/23,343 (0.3%)	15/3909 (0.4%)	7/3640 (0.2%)	4/3274 (0.1%)	17/4869 (0.3%)	13/4093 (0.3%)	7/3558 (0.2%)	
Motorcycle ambulance	69/23,343 (0.3%)	15/3909 (0.4%)	9/3640 (0.2%)	33/3274 (1.0%)	12/4869 (0.2%)	0/4093 (0.0%)	0/3558 (0.0%)	
ALS ambulance	2226/23,343 (9.5%)	369/3909 (9.4%)	304/3640 (8.4%)	269/3274 (8.2%)	586/4869 (12.0%)	410/4093 (10.0%)	288/3558 (8.1%)	
BLS ambulance	20,979/23,343 (89.9%)	3510/3909 (89.8%)	3318/3640 (91.2%)	2966/3274 (90.6%)	4254/4869 (87.4%)	3669/4093 (89.6%)	3262/3558 (91.7%)	
Rapid intervention vehicle	6/23,343 (0.0%)	0/3909 (0.0%)	2/3640 (0.1%)	2/3274 (0.1%)	0/4869 (0.0%)	1/4093 (0.0%)	1/3558 (0.0%)	
**Reason for EMS call**								**<0.001**
Motor vehicle collisions	3521/31,455 (11.2%)	691/5114 (13.5%)	759/4903 (15.5%)	608/5385 (11.3%)	262/5346 (4.9%)	446/5612 (7.9%)	755/5095 (14.8%)	
Dead at scene	616/31,455 (2.0%)	105/5114 (2.1%)	84/4903 (1.7%)	77/5385 (1.4%)	120/5346 (2.2%)	142/5612 (2.5%)	88/5095 (1.7%)	
Aggression/SW/GSW	1862/31,455 (5.9%)	392/5114 (7.7%)	364/4903 (7.4%)	285/5385 (5.3%)	170/5346 (3.2%)	305/5612 (5.4%)	346/5095 (6.8%)	
Other Trauma	2211/31,455 (7.0%)	431/5114 (8.4%)	479/4903 (9.8%)	379/5385 (7.0%)	207/5346 (3.9%)	291/5612 (5.2%)	424/5095 (8.3%)	
Surgical	343/31,455 (1.1%)	75/5114 (1.5%)	63/4903 (1.3%)	39/5385 (0.7%)	47/5346 (0.9%)	54/5612 (1.0%)	65/5095 (1.3%)	
Cardiac emergencies	2063/31,455 (6.6%)	395/5114 (7.7%)	359/4903 (7.3%)	323/5385 (6.0%)	268/5346 (5.0%)	363/5612 (6.5%)	355/5095 (7.0%)	
Other medical emergencies *	4505/31,455 (14.3%)	582/5114 (11.4%)	572/4903 (11.7%)	840/5385 (15.6%)	1171/5346 (21.9%)	688/5612 (12.3%)	652/5095 (12.8%)	
Respiratory emergencies	4935/31,455 (15.7%)	447/5114 (8.7%)	398/4903 (8.1%)	709/5385 (13.2%)	1335/5346 (25.0%)	1464/5612 (26.1%)	582/5095 (11.4%)	
Neurological emergencies	2611/31,455 (8.3%)	472/5114 (9.2%)	475/4903 (9.7%)	458/5385 (8.5%)	346/5346 (6.5%)	380/5612 (6.8%)	480/5095 (9.4%)	
Gastrointestinal emergencies	1669/31,455 (5.3%)	299/5114 (5.8%)	286/4903 (5.8%)	279/5385 (5.2%)	253/5346 (4.7%)	262/5612 (4.7%)	290/5095 (5.7%)	
Ob/Gyn emergencies	525/31,455 (1.7%)	101/5114 (2.0%)	105/4903 (2.1%)	97/5385 (1.8%)	51/5346 (1.0%)	75/5612 (1.3%)	96/5095 (1.9%)	
Mental health emergencies	1243/31,455 (4.0%)	227/5114 (4.4%)	255/4903 (5.2%)	190/5385 (3.5%)	136/5346 (2.5%)	219/5612 (3.9%)	216/5095 (4.2%)	
Intoxication	494/31,455 (1.6%)	105/5114 (2.1%)	110/4903 (2.2%)	80/5385 (1.5%)	31/5346 (0.6%)	84/5612 (1.5%)	84/5095 (1.6%)	
Occupational accidents	8/31,455 (0.0%)	3/5114 (0.1%)	1/4903 (0.0%)	0/5385 (0.0%)	0/5346 (0.0%)	2/5612 (0.0%)	2/5095 (0.0%)	
Animal bites	262/31,455 (0.8%)	75/5114 (1.5%)	62/4903 (1.3%)	46/5385 (0.9%)	19/5346 (0.4%)	23/5612 (0.4%)	37/5095 (0.7%)	
Others	4587/31,455 (14.6%)	714/5114 (14.0%)	531/4903 (10.8%)	975/5385 (18.1%)	930/5346 (17.4%)	814/5612 (14.5%)	623/5095 (12.2%)	
**Type of call**								**<0.001**
External causes	7929/34,979 (22.7%)	1612/5714 (28.2%)	1681/5415 (31.0%)	1337/6006 (22.3%)	651/6057 (10.7%)	1083/6101 (17.8%)	1565/5686 (27.5%)	
Surgical	125/34,979 (0.4%)	15/5714 (0.3%)	25/5415 (0.5%)	16/6006 (0.3%)	13/6057 (0.2%)	17/6101 (0.3%)	39/5686 (0.7%)	
Medical	19,540/34,979 (55.9%)	2883/5714 (50.5%)	2645/5415 (48.8%)	3186/6006 (53.0%)	4143/6057 (68.4%)	3725/6101 (61.1%)	2958/5686 (52.0%)	
Ob/Gyn	848/34,979 (2.4%)	166/5714 (2.9%)	163/5415 (3.0%)	150/6006 (2.5%)	96/6057 (1.6%)	124/6101 (2.0%)	149/5686 (2.6%)	
Not evaluated	4501/34,979 (12.9%)	703/5714 (12.3%)	522/5415 (9.6%)	952/6006 (15.9%)	918/6057 (15.2%)	799/6101 (13.1%)	607/5686 (10.7%)	
Pediatrics	705/34,979 (2.0%)	82/5714 (1.4%)	107/5415 (2.0%)	160/6006 (2.7%)	100/6057 (1.7%)	124/6101 (2.0%)	132/5686 (2.3%)	
Mental health	1331/34,979 (3.8%)	253/5714 (4.4%)	272/5415 (5.0%)	205/6006 (3.4%)	136/6057 (2.2%)	229/6101 (3.8%)	236/5686 (4.2%)	
**Response time (minutes)**	35.0 (24.0–54.6)	32.8 (21.8–48.1)	32.8 (21.8–48.1)	32.8 (21.8–48.1)	45.9 (30.6–67.7)	43.7 (28.4–63.4)	35.0 (24.0–50.2)	**<0.001**
**City zones**								0.88
West central	2423/33,670 (7.2%)	390/5455 (7.1%)	361/5208 (6.9%)	406/5789 (7.0%)	407/5866 (6.9%)	448/5896 (7.6%)	411/5456 (7.5%)	
South central	2945/33,670 (8.7%)	470/5455 (8.6%)	509/5208 (9.8%)	587/5789 (10.1%)	474/5866 (8.1%)	435/5896 (7.4%)	470/5456 (8.6%)	
East	7498/33,670 (22.3%)	1249/5455 (22.9%)	1096/5208 (21.0%)	1321/5789 (22.8%)	1325/5866 (22.6%)	1332/5896 (22.6%)	1175/5456 (21.5%)	
North	9706/33,670 (28.8%)	1578/5455 (28.9%)	1487/5208 (28.6%)	1596/5789 (27.6%)	1647/5866 (28.1%)	1786/5896 (30.3%)	1612/5456 (29.5%)	
West	4544/33,670 (13.5%)	681/5455 (12.5%)	673/5208 (12.9%)	755/5789 (13.0%)	849/5866 (14.5%)	833/5896 (14.1%)	753/5456 (13.8%)	
South	6385/33,670 (19.0%)	1068/5455 (19.6%)	1068/5208 (20.5%)	1101/5789 (19.0%)	1135/5866 (19.3%)	1017/5896 (17.2%)	996/5456 (18.3%)	
Riverine communities	169/33,670 (0.5%)	19/5455 (0.3%)	14/5208 (0.3%)	23/5789 (0.4%)	29/5866 (0.5%)	45/5896 (0.8%)	39/5456 (0.7%)	

Abbreviations and symbols: ALS, advanced life support; BLS, basic life support; GSW, gunshot wound; SW, stab wound; Ob/Gyn, obstetrics and gynecology. * pain, edema, epistaxis, hemorrhage, hyperglycemia, hypothermia, infection, eye complaints, neoplasm, hypoactivity, and bleeding.

**Table 3:** Contrast of transports by the prehospital EMS in Manaus, before and after the first confirmed case of COVID-19 in the state of Amazonas (13 March 2020).

	Total	Pre-Pandemic	Pandemic	*p*-Value
	*n* = 45,581	*n* = 14,133	*n* = 31,448	
**Age (years)**	47.0 (30.0–67.0)	44.0 (27.0–66.0)	48.0 (30.0–68.0)	**<0.001**
**Dispatched unit**				**0.0007**
Boat ambulance	63/23,343 (0.3%)	25/8888 (0.3%)	38/14,455 (0.3%)	
Motorcycle ambulance	69/23,343 (0.3%)	34/8888 (0.4%)	35/14,455 (0.2%)	
ALS ambulance	2226/23,343 (9.5%)	762/8888 (8.6%)	1464/14,455 (10.1%)	
BLS ambulance	20,979/23,343 (89.9%)	8064/8888 (90.7%)	12,915/14,455 (89.3%)	
Rapid intervention vehicle	6/23,343 (0.0%)	3/8888 (0.0%)	3/14,455 (0.0%)	
**Reason for EMS call**				**<0.001**
Motor vehicle collision	3521/31,455 (11.2%)	1770/11,887 (14.9%)	1751/19,568 (8.9%)	
Death at scene (non-traumatic)	616/31,455 (2.0%)	208/11,887 (1.7%)	408/19,568 (2.1%)	
Physical aggression/GSW/SW	1862/31,455 (5.9%)	867/11,887 (7.3%)	995/19,568 (5.1%)	
Other traumatic emergencies	2211/31,455 (7.0%)	1055/11,887 (8.9%)	1156/19,568 (5.9%)	
Surgical emergencies	343/31,455 (1.1%)	152/11,887 (1.3%)	191/19,568 (1.0%)	
Cardiac emergencies	2063/31,455 (6.6%)	878/11,887 (7.4%)	1185/19,568 (6.1%)	
Other medical emergencies	4505/31,455 (14.3%)	1356/11,887 (11.4%)	3149/19,568 (16.1%)	
Respiratory emergencies	4935/31,455 (15.7%)	991/11,887 (8.3%)	3944/19,568 (20.2%)	
Neurological emergencies	2611/31,455 (8.3%)	1138/11,887 (9.6%)	1473/19,568 (7.5%)	
Gastrointestinal emergencies	1669/31,455 (5.3%)	689/11,887 (5.8%)	980/19,568 (5.0%)	
Ob/Gyn emergencies	525/31,455 (1.7%)	248/11,887 (2.1%)	277/19,568 (1.4%)	
Mental health emergencies	1243/31,455 (4.0%)	559/11,887 (4.7%)	684/19,568 (3.5%)	
Intoxication	494/31,455 (1.6%)	255/11,887 (2.1%)	239/19,568 (1.2%)	
Occupational accidents	8/31,455 (0.0%)	4/11,887 (0.0%)	4/19,568 (0.0%)	
Animal bites	262/31,455 (0.8%)	149/11,887 (1.3%)	113/19,568 (0.6%)	
Others	4587/31,455 (14.6%)	1568/11,887 (13.2%)	3019/19,568 (15.4%)	
**Type of call**				**<0.001**
External cause	7929/34,979 (22.7%)	3892/13,197 (29.5%)	4037/21,782 (18.5%)	
Surgical	125/34,979 (0.4%)	46/13,197 (0.3%)	79/21,782 (0.4%)	
Medical	19,540/34,979 (55.9%)	6497/13,197 (49.2%)	13,043/21,782 (59.9%)	
Ob/Gyn	848/34,979 (2.4%)	392/13,197 (3.0%)	456/21,782 (2.1%)	
Not evaluated	4501/34,979 (12.9%)	1534/13,197 (11.6%)	2967/21,782 (13.6%)	
Pediatrics	705/34,979 (2.0%)	231/13,197 (1.8%)	474/21,782 (2.2%)	
Mental health	1331/34,979 (3.8%)	605/13,197 (4.6%)	726/21,782 (3.3%)	
**Response time (minutes)**	35.0 (24.0–54.6)	32.8 (21.8–48.1)	39.3 (26.2–59.0)	**<0.001**
**City zones**				0.80
West central	2423/33,670 (7.2%)	889/12,637 (7.0%)	1534/21,033 (7.3%)	
South central	2945/33,670 (8.7%)	1170/12,637 (9.3%)	1775/21,033 (8.4%)	
East	7498/33,670 (22.3%)	2784/12,637 (22.0%)	4714/21,033 (22.4%)	
North	9706/33,670 (28.8%)	3632/12,637 (28.7%)	6074/21,033 (28.9%)	
West	4544/33,670 (13.5%)	1594/12,637 (12.6%)	2950/21,033 (14.0%)	
South	6385/33,670 (19.0%)	2522/12,637 (20.0%)	3863/21,033 (18.4%)	
Riverine communities	169/33,670 (0.5%)	46/12,637 (0.4%)	123/21,033 (0.6%)	
